# Status of Extended Threshold Wavelength Split-Off Band IR Detectors and Quantum Material-Based Extension for Room-Temperature Operation

**DOI:** 10.3390/mi16030286

**Published:** 2025-02-28

**Authors:** A. G. Unil Perera, Yanfeng Lao, Tara Jabegu, Sidong Lei

**Affiliations:** 1 Department of Physics and Astronomy, Georgia State University, Atlanta, GA 30303, USA; ylao95@gmail.com (Y.L.); tjabegu1@gsu.edu (T.J.); sidong.lei@ucf.edu (S.L.); 2 Hisense Photonics Inc., South Plainfield, NJ 07080, USA; 3 NanoScience Technology Center, University of Central Florida, Orlando, FL 32826, USA; 4 Department of Materials Science and Engineering, University of Central Florida, Orlando, FL 32816, USA

**Keywords:** IR detectors, split-off band IR detectors, high-temperature IR detectors, high performance IR sensing, quantum IR detectors

## Abstract

This paper reports possible performance improvements of split-off band infrared detectors by using novel quantum materials. The report starts by describing the development of split-off band infrared detectors based on heterostructures with extended photoresponsivity beyond the energy band gap. The design modification demonstrated a new phenomenon of extending the threshold wavelength beyond the standard wavelength threshold (*λ*_t_) determined by the energy gap (Δ) and the wavelength equation *λ*_t_ = 1.24/Δ with the dark current still governed by the original energy gap. However, to overcome the operating temperature challenges in AlGaAs/GaAs-based devices, the perspective of van der Waals quantum materials (vdW-QM)-based IR sensors is discussed regarding the aspects of heterostructure fabrication methods, theoretical modeling, and strategies that could help to overcome these issues. Through these discussions, the review paper aims to inspire upcoming innovations in developing novel IR photodetectors capable of operating within the atmospheric window at room temperature.

## 1. Introduction

Conventional photodetection devices usually rely on interband excitation. Therefore, narrow-gap semiconductors, such as HgCdTe, are dominating materials for infrared (IR) photodetector fabrication [1,2,3,4]. However, narrow band-gap semiconductors accompany interband thermal excitation and noise; thus, they require cryocoolers to obtain the desired sensitivity and detectivity. Due to this intrinsic limitation in conventional IR sensing mechanisms, the research focus has shifted to the construction of new device configurations and work mechanisms, such as quantum wells and Type II superlattice detectors [5,6,7,8,9,10,11,12,13,14]. These structures can potentially separate the photoresponse and thermal excitation processes, in order to provide sufficient detectivity in the atmospheric window at room temperature.

**Figure 1:**
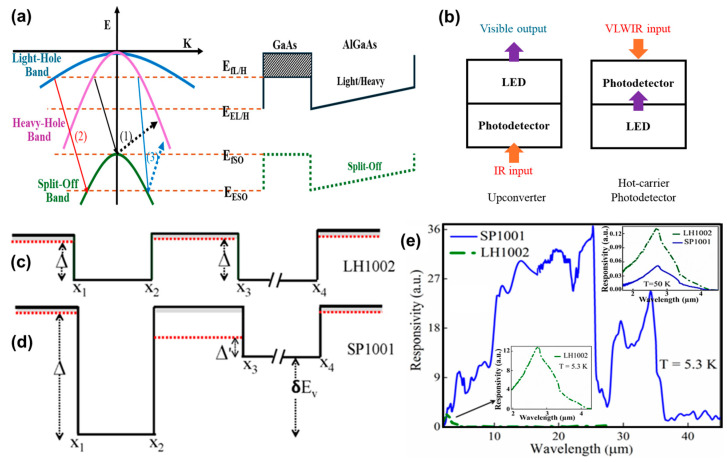
(**a**) Energy band structure and spin–orbit splitting (Δ_SO_) of GaAs showing split-off detector threshold mechanisms, where the IR photon excites holes from the light/heavy hole bands to the split-off band. (1) Indirect absorption followed by scattering and escape (threshold energy: *E*_fSO_ − *E*_fL/H_). (2) Direct absorption followed by scattering and escape (threshold energy: *E*_ESO_ − *E*_fL/H_). (3) Indirect absorption followed by escape and some scattered (threshold energy: *E*_fSO_ − *E*_fL/H_). (**b**) Two examples of split-off detector threshold mechanism applications, an upconverter and a hot-carrier photodetector. (**c**,**d**) Valence band alignment of Al_x_Ga_1−x_As IR split-off detectors with symmetric and asymmetric heterojunction structures serving as hole barriers. (**e**) Photoresponsivity spectrum of the IR detectors. The asymmetric heterojunction shows spectral extension from a short wavelength (corresponding to Δ) to mid- to long-IR range due to the quasi-Fermi level of Δ′.

Perera et al. [15,16,17] demonstrated an IR detector operating at 130 K using a GaAs/AlGas heterostructure with a threshold wavelength of ~20 μm, producing a split-off response in the range of 1.5–5 μm with a peak specific detectivity (D*) of 1.0 × 10^8^ Jones. Device modeling [18] predicted a graded barrier design with an offset to have a much better performance. Using a design based on the model resulted in a wavelength threshold extension beyond the standard threshold, extending the IR sensing [15] with suppressed thermal noise in GaAs/AlGaAs heterostructures beyond the expected design wavelength given by the energy gap (Δ). By using the split-off bands in AlGaAs-based heterostructures (see Figure 1a), an intra-band, instead of interband, photoexcitation [15,16,17] could be successfully achieved, inducing split-off band based intra-band absorption and photoresponse [17], showcasing a 3~5 µm IR response in the first atmospheric window at room temperature [19]. This mechanism can be applied to the detectors as shown in Figure 1b. A similar up-conversion idea was demonstrated by Liu et al. [20] and more recently additional up-conversion ideas are reported elsewhere [21]. Furthermore, going beyond symmetric valence band alignment (Figure 1c) by introducing an asymmetric valence band alignment in the heterostructure (Figure 1d, which only shows the valence bands) [22,23], the photoresponsivity spectral threshold wavelength can be further extended to a longer wavelength (Figure 1e) [22], thanks to a quasi-Fermi level (denoted by Δ′ in Figure 1d) formed in the absorber layers (which is the x_2_-x_3_ region) [24,25,26]. This quasi-Fermi level is created by the hot holes traveling through the barrier layer (x_1_-x_2_ region in Figure 1d) and elastically injected into the absorber layers. The holes in the emitter with high energies remain in the heavy-hole band and escape from the same band after absorbing a low-energy photon. The split-off band is not involved in this process and therefore has no impact on the extension response. The mechanism for the extension was understood by performing a pump-probe experiment. A pump light (with high-energy photons) is introduced to generate hot holes, and the probe light is used to detect the response, which has an energy lower than the response threshold of the absorber. The details can be found in Reference [15]. In contrast, the symmetric structure shown in Figure 1c did not exhibit such an effect. More interestingly, this spectral extension did not increase the dark current level, which was still blocked by the height of the hole injection barrier (Δ) instead of Δ′ [15,27]. Under dark conditions, since there is no injection of hot holes and the current results from thermionic emission, the major factors that affect the currents are the potential barrier and the hole distribution. The dark current can be largely affected by the potential barrier that carriers come across. A consequence due to the gradient could be the increased probability of hole tunnelling through the triangle barrier which increases the dark current in addition to the thermionic effect. However, the potential barrier Δ still dominantly affects the dark current. In such a manner, photoexcitation and thermal excitation can be successfully separated and provide a feasible pathway towards room-temperature IR sensing in the atmospheric window. To improve the detector performance, absorption efficiency and the photocarrier escape rate can be two key factors for enhancement. Absorption can be significantly increased by employing multiple emitters instead of the single-emitter structure (Figure 1c,d). Optical cavity enhancement creates multiple passes through the emitter by incorporating top and bottom reflecting layers, further increasing absorption efficiency. Regarding the photocarrier escape rate, escape occurs as photocarriers are transported over the potential barrier at the GaAs/AlGaAs interface. A device model as shown in Reference [18] provides a feasibility study of coupling holes from the emitter’s energy band to that of the barrier. Although Reference [18] discusses coupling between the split-off band and the heavy-hole band, the same concept can be applied to heavy-hole band coupling. The quantum efficiency can also be further enhanced by utilizing 2D material-based IR sensors. Few-layered InSe exhibits an external quantum efficiency (EQE) of approximately 8% [28]. Considering that the absorption rate is typically below 10% [29,30], the internal quantum efficiency (IQE) can exceed 90%. The EQE can be enhanced by approximately 900% through the incorporation of plasmonic antennas, demonstrating that a high EQE can also be achieved under optimized conditions [31]. By incorporating the graded barrier and the offset, a long-wavelength photovoltaic response (up to 8 µm) in a short-wavelength-type GaAs heterojunction detector (with the activation energy of E_A_ = 0.40 eV) operating at 80 K was demonstrated. This wavelength-extended photovoltaic response is enabled by employing a non-symmetrical band alignment. The detectivity (D*) at 5 um was obtained to be 3.5 × 10^12^ Jones, an improvement by a factor of 10^5^ over the detector without the wavelength extension.

Beyond that, novel two-dimensional (2D) quantum materials structures, such as bi-layer graphene with twisted angles, black phosphor structures, and transition metal dichalcogenide (TMDCs), also exhibited adjustable IR responses [32,33,34]. As such, these emerging materials and structures can potentially enhance the above-mentioned IR sensing mechanism and enrich the selection of materials, providing more flexibility in device design and applications.

In this review paper, we insightfully summarize the working principles of AlGaAs-based IR detectors and their limitations, including (1) a short hot-hole lifetime that limits operating temperatures and (2) a narrow adjustable Al-to-Ga ratio that restricts the tunable ranges of Δ and Δ′, thus impeding wavelength extension and the optimization of the dark current level, photoresponsivity, detectivity, and other crucial properties for practical applications. Then, we will discuss the possibility of reassembling the AlGaAs heterostructure with 2D material stacking structures using ultra-clean and flat heterostructure interfaces, less charge carrier scattering, and no lattice matching restriction. This discussion can inspire the following exploration for better IR sensing architectures and help to verify the versatility of the IR sensing mechanism beyond the AlGaAs system.

## 2. Benchmarks of Split-Off Band IR Detection

Perera et.al demonstrated the split-off band IR detector by using hole transitions from the light/heavy hole bands (LH/HH) to the spin–orbit split-off (SO) band [16,17] with the photo carriers escaping over the AlGaAs barrier through an internal photoemission process, as illustrated in Figure 1a. The split-off detector with 30 periods of 3 × 10^18^ cm^−3^ p-doped 18.8 nm GaAs emitters and 60 nm undoped Al_0.57_Ga_0.43_As barriers were measured. A peak responsivity of 0.29 mA/W, and a peak detectivity of (D^∗^) ~ 6.8 × 10^5^ Jones were observed at 2.5 μm at room temperature. In a sealed enclosure the detector continuously operated for several weeks (until stopped) with an equilibrium temperature at 330 K. In addition, the hole transitions from heavy-hole to light-hole bands contributed to the long-wavelength spectral response extending beyond 14 μm (Figure 1e), as observed in a p-GaAs/Al_0.28_Ga_0.72_ As heterostructure. Two characteristic peaks observed between 5 and 7 μm are in good agreement with the LH and HH bands splitting energies. Table 1 lists the key parameters of several detectors with a symmetric configuration shown in Figure 1c.

The dark current of these IR photodetectors is described by a 3D carrier drift model [27,35] governed by Δ as expressed in Equation (1) [15,27,37].

(1) Idark=2AevFm∗kBT2πℏ232exp⁡−Δ−αFkBT

where *A* is the electrically active area of the detector, *e* is the electronic charge,$\;v\left(F\right)$ is the carrier drift velocity as a function of the electric field, $m^{*}$ is the effective mass, $k_{B}$ is Boltzmann’s constant, *T* is the temperature, ℏ is the reduced Planck’s constant, and α is a fitting parameter that determines the effective barrier lowering due to the applied field [27].

**Figure 2:**
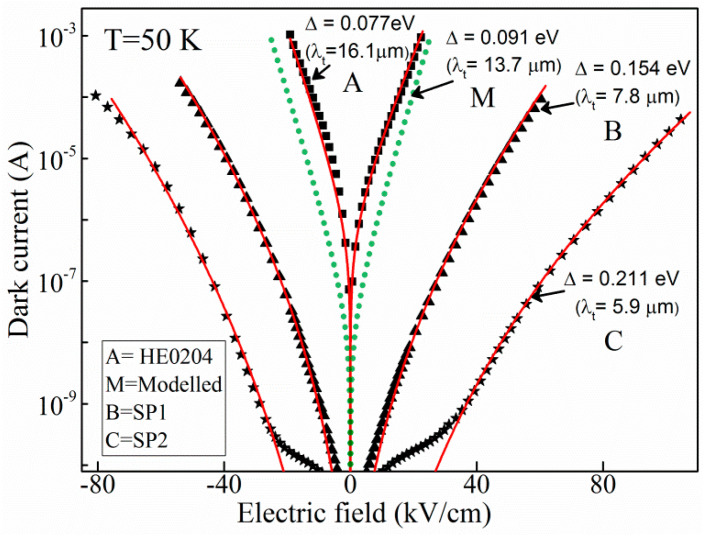
Experimental and 3D model dark current for the samples HE0204 (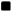), SP1 (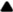), and SP2 (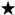). In addition, a simulated dark current for a modeled detector (•••) of 13.7 μm using a 3D drift model clearly indicating the agreement of the experimental dark current with the model results.

Figure 2 shows the experimental dark current curves of the devices listed in Table 1 (HE0204 (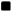), SP1 (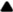), and SP2 (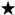)) and the fitting results (solid red lines) by using Equation (1). With the experimental data of λ_t_, λ_eff_, and Δ for HE0204, SP1, and SP2, (16.1 µm, 8.2 µm, and 6.0 µm, respectively) substituted, the fitting curves shows excellent agreement with the experimental observations. A simulated dark current for a modeled symmetric detector (labeled as M) of Δ = 0.091 eV (13.7 µm) is also shown by the dotted green (•••) line in Figure 2. The dark current data for the sample LH1002 and the photoresponse spectra for listed samples (Table 1) are available in earlier reports [1,2,3,38]. This analysis indicates that the 3D carrier drift model can successfully explain the dark current behavior of the symmetric AlGaAs heterostructures.

### 2.1. Effect of the Barrier Energy Offset (with Flat Injector Barrier)

More interestingly, an extended IR response was observed when the symmetric structure was replaced by an asymmetric counterpart. The asymmetric structures feature a barrier energy offset labeled as δEv in Figure 1d. Table 2 lists the key parameters of several asymmetric devices.

**Figure 3:**
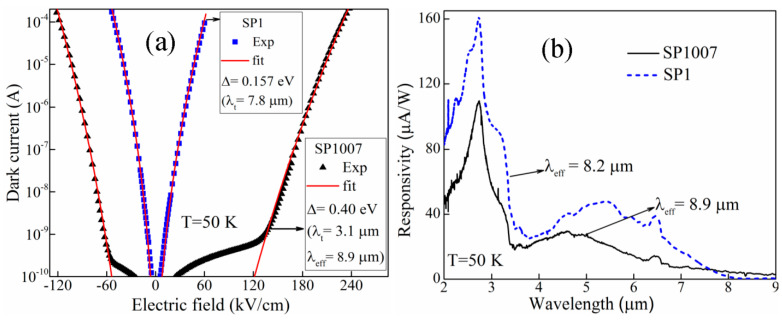
(**a**) Drift model dark current (solid red line) and experimental dark current of symmetric [SP1 (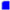)] and asymmetric [SP1007 (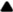)] GaAs-based split-off IR sensors showing a clear difference in dark currents between the two structures. (**b**) The response spectra of SP1007 shows λ_eff_ = 8.9 μm (Δ′ = 0.139 eV) < Δ = 0.40 eV (λ_t_ = 3.1 μm) obtained from the dark current, whereas for SP1, λ_eff_ = 8.2 μm (Δ′ = 0.151 eV) matches with the value of Δ = 0.154 eV obtained from dark current fitting.

The dark currents of the device SP1 (symmetric) and the device SP1007 (asymmetric) are compared in Figure 3. It can be found that SP1007 shows a much lower dark current than SP1, even though it has a longer threshold wavelength. The phenomenon clearly demonstrates that the Δ value controls the dark current, while the effective Δ′ controls the threshold wavelength [38,39]. The comparison between a symmetric detector (LH1002) and an asymmetric detector (SP1001) shows a similar trend, as illustrated in Figure 1e. Even though the Δ value suggests a cutoff wavelength of 3.1 µm, SP1001 shows a λ_eff_ of ~36 µm at 5.3 K, and 4.1 µm at 50 K (inset of Figure 1e). This can be explained with the following argument. Both the symmetric and asymmetric IR sensors rely on the photoexcitation between the GaAs split-off bands [27,40]. In the symmetry configuration, no photoresponse is observed with a zero bias electric field; with an electric field, a spectral response with threshold wavelength λ_t_ = 1.24/Δ is observed. In contrast, when such symmetry is broken (Figure 1d), the hot holes generated by short-wavelength radiation inject into the absorber and form a quasi-Fermi level (Δ′), which in turn defined another threshold wavelength of λ_eff_ = 1.24/Δ′ that was much longer than λ_t_. This is the fundamental principle of the extended IR response in the asymmetric heterojunction IR detectors. Figure 1e demonstrates that the symmetric device (LH1002) shows a threshold wavelength of 3.1 µm, whereas the asymmetric design (SP1001) extends the response up to ~36 µm (at 5.3 K). Because Δ >> Δ′, the dark current can be effectively suppressed while maintaining the desired threshold response wavelength [26]. Hence, asymmetric heterostructures bring another essential benefit besides the extension of the IR response range, intangible in any other IR photosensors. The quasi-Fermi level is formed by hot holes deviating from thermal equilibrium, as such separating the photoexcitation and thermal excitation. Therefore, the extended IR response range does not necessarily accompany a higher dark current as in the symmetric conventional detectors.

### 2.2. Advantages in Dark Current and D* with Increased Barrier Energy Offset

**Figure 4:**
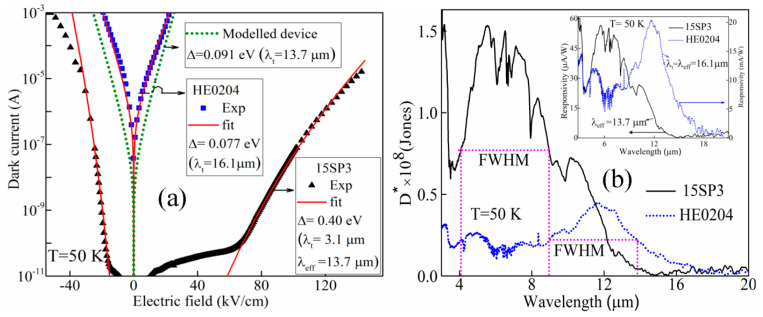
(**a**) Comparison of dark current for HE0204 (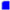) and 15SP3 (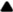), showing a clear difference in dark current for both structures for a similar λ_t_. A dotted green (•••) line shows a simulated dark current of a modeled detector with Δ = 0.091 eV (λ_t_ = 13.7 μm). (**b**) D* for HE0204 and 15SP3, clearly showing a higher D* for 15SP3; the inset shows the response of HE0204, λ_eff_ = 16.1 μm (Δ′ = 0.077 eV) which was fitted to the dark current with Δ = 0.077 eV; for 15SP3, Δ′ (λ_eff_) is 0.091 eV (13.7 μm) fitted to the dark current with Δ = 0.40 eV corresponding to λ_t_ = 3.1 μm.

In line with the reduced (separately controlled) dark current, the detectivity (D*) of devices also improved. To demonstrate both the dark current and D* advantages, 15SP3 has a δE_v_ further increased to 0.19 eV. Its dark current level and D* are compared with a regular detector (HE0204), as shown in Figure 4a,b. The dark current fit for a model detector of Δ = 0.091 eV (λ_t_ =13.7 µm) is also shown by the green dotted (••) line in Figure 4a. For device HE0204, the Δ value is set at 0.077 eV for fitting, corresponding to λ_t_ = 16.1 µm, which matches the experimentally determined threshold wavelength. In contrast, the dark current in 15SP3 can be fitted with Δ ~0.40 eV, i.e., λ_t_ = 3.1 µm, whereas the experimental λ_eff_ has a value of 13.7 µm (i.e., Δ′ = 0.091 eV), as indicated by Figure 4b. Again, this confirms that the dark current and threshold wavelength are adjusted independently. The specific detectivities (D*) for both samples are shown in Figure 4b, where the D* (both peak and FWHM) is higher in 15SP3 as compared to the conventional device HE0204. The inset of Figure 4b shows the responsivities of HE0204 and 15SP3.

### 2.3. Effect of the Gradient of the Injector Barrier (with the Same Barrier Energy Offset)

In addition to the barrier energy offset, the barrier shape also plays an essential role in determining dark current, threshold wavelength, and detectivity. In particular, by adjusting the barrier shape, it is possible to obtain a better high-temperature device performance.

**Figure 5:**
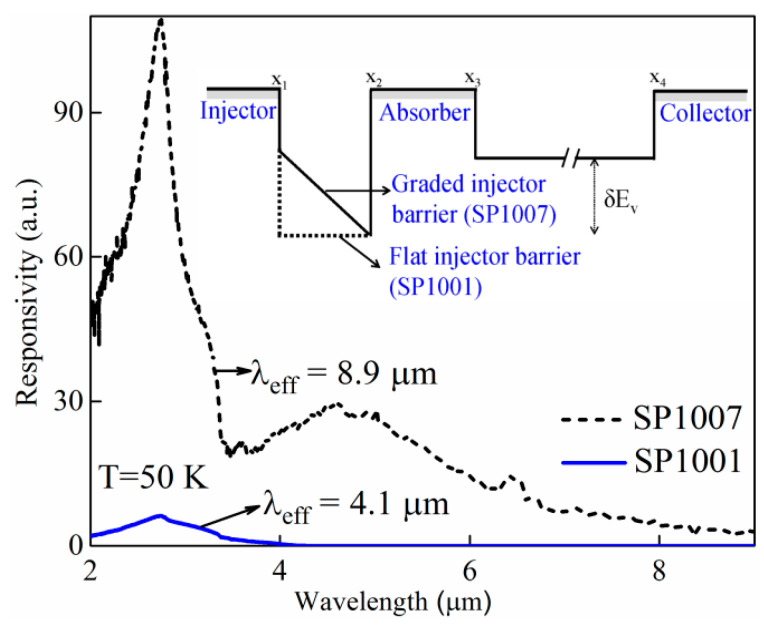
Response spectra of SP1001 and SP1007: as the injector barrier changes from flat to graded, the λ_eff_ increases from 4.1 μm to 8.9 μm.

As mentioned above, the threshold wavelength of SP1001 reduces from ~36 µm to 4.1 µm, as the temperature increases from 5.3 K to 50 K. In other words, at a higher temperature, λ_eff_ approaches λ_t_ (see Table 2), indicating the disappearance of the quasi-Fermi level. As a comparison, the device SP1007 with a graded barrier configuration, as illustrated in Figure 5 inset, exhibits a much better high-temperature performance. Although SP1007 has the same barrier energy offset (δEv) as SP1001, its λ_eff_ is much longer than that of SP1001, suggesting the persistence of the quasi-Fermi level at 50 K. This implies that by adjusting the gradient of the injector barrier, the λ_eff_ can be increased (Δ′ decreased) leading to a wider detection range. A possible explanation is that the graded barrier reduces the hot-carrier traveling path. Therefore, with the same lifetime, more hot holes can be injected into the absorption layer and build the quasi-Fermi level.

## 3. An Empirical Model for Hot-Hole Effects in the IR Sensor

**Figure 6:**
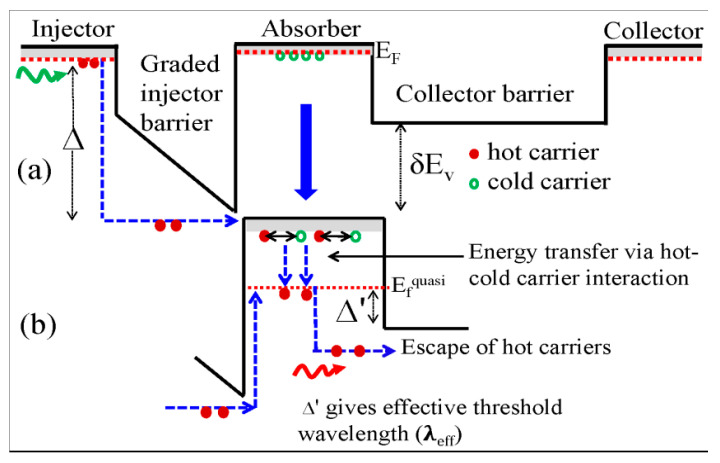
(**a**) Unbiased schematic VB diagram of graded injector barrier with barrier energy offset. E_f_ is the Fermi level at a lattice temperature (T_L_); upon photoexcition, hot holes surmount the graded injector barrier and interact with cold holes in the absorber. For clarity, the absorber section is separated. The solid (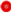) dots and empty (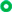) dots represent hot holes and cold holes in the absorber, and the green wavy arrow represents an incident photon with an energy exceeding Δ, whilst the red wavy arrow in the absorber shows an incident photon with an energy of Δ′. (**b**) Energy transfer via hot hole–cold hole interaction and the formation of a quasi-Fermi level (E_f_^quasi^) at a hot-hole temperature greater than the lattice temperature finally leads to the escape of hot holes from that E_f_^quasi^ by the absorption of a long-wavelength photon, giving Δ′.

The dark current advantage described in connection with λ_eff_ (Δ′) in the asymmetric detectors can be explained as the hot-cold carrier interaction in the absorber [15,27,41]. The valence band diagram of the IR sensor is shown in Figure 6a. E_f_ is the Fermi level at a lattice temperature (T_L_) and E_f_^quasi^ is the quasi-Fermi level at a hot-carrier temperature (T_c_) (the absorber section is highlighted in Figure 6b for clarity). With the incident of IR photons, hot holes with an energy > Δ will surmount the graded injector barrier and interact with cold holes in the absorber (the contribution from the collector side is ignored in this discussion).

At zero bias, a net flow of hot carriers from the graded injector to the collector barrier will be observed owing to the difference in barrier heights. A higher δE_v_ and the gradient of the injector barrier will increase the flow. Some fraction of hot-hole energy is transferred to the cold hole (in the absorber), leading to non-equilibrium carrier densities with specific momentum states and elevated carrier temperature. Carrier–carrier scattering results in Coulomb thermalization and allows the carrier system to be described by a quasi-Fermi Dirac distribution with a temperature of T_c_ >> T_L_. As a long-wavelength (λ_eff_) photon with an energy greater than (Δ′) is absorbed at this quasi-Fermi level (E_f_^quasi^), hot holes will escape and be collected across the collector barrier. Similar to the case in a conventional detector, with increasing bias (up to a certain limit), the energy of holes passing over the graded barrier increases, leading to a greater transfer of energy to the cold holes in the absorber and in turn increasing the number of hot holes in the absorber. Hence, the number of hot holes escaping from the E_f_^quasi^ over the collector barrier also increases, thereby increasing the response strength of the extended wavelength photoresponse.

To calculate the escape probability of hot carriers, an escape cone model with E_f_^quasi^ will be used. The responsivity [27] (R) of the detector depends on the total quantum efficiency (*η*), electron charge (q), speed of light in vacuum (c), and Planck’s constant (h) as given by

(2) R=qηλhc

The total quantum efficiency (*η*) is the product of the photon absorption probability (*η*_a_), the internal quantum efficiency (*η*_i_), and the hot-carrier transport probability (*η*_t_) and is given as

(3) $$\eta =\eta_{a}\eta_{i}\eta_{t}$$

The values of *η*_a_ and *η*_t_ were calculated using a model described elsewhere [27,42]. η_i_ was calculated using an escape cone model [40], and the calculation of *η*_i_ can take into account the scattering of hot holes with cold holes as well as phonons, given as follows [43]:

(4) $$\eta_{i}=\eta_{0}+\left[1-\frac{\eta_{0}}{\eta_{M}}\right]\gamma \eta_{1}+\left[1-\frac{\eta_{0}}{\eta_{M}}\right]\left[1-\frac{\eta_{1}}{\eta_{M}}\right]\gamma^{2}\eta_{1}+\cdots$$

where $\eta_{n}=\eta_{0}\left(E-nh\nu \right)$ and $\gamma =L_{h}/\left(L_{e}+L_{h}\right)$, *n* is the number of scattering events, *η*_M_ is the maximum quantum efficiency, and the value of *η*_0_ is defined as the fraction of hot holes captured prior to any bulk scattering events and is given by

(5) $$\eta_{0}=\frac{L^{*}}{W}{\left(1-e^{-W/L^{*}}\right)}^{1/2}\eta_{Ideal}$$

where W is the width of the absorber, and *η*_ideal_ is the ideal quantum efficiency. $L^{*}=L_{h}\times L_{p}/\left(L_{h}+L_{p}\right)$ is the reduced scattering length of hot hole–cold hole, L_h_ is the hole–hole scattering length for hot hole–cold hole, and L_p_ represents the elastic scattering of hot holes with phonons and impurities, as well as the multiple reflections of the excited hot holes from the surfaces of the absorber. The value of E_f_^quasi^ can be determined by the difference between the valence band edge (ΔE_v_) and Δ′ as determined by the TDIPS [26] fitting of photoresponse spectra.

## 4. Challenges of Visions

Despite the advantages in terms of higher operating temperature, lower dark current, and correspondingly, better detectivity, the AlGaAs-base IR detectors with an extended threshold wavelength are still not operating at room temperature. This is because the quasi-Fermi level (Δ′) gradually disappears and λ_eff_ approaches λ_t_ as defined by the split-off bands when the temperature rises. Several reasons could impede these performance improvements.

1. In the Al_x_Ga_1-x_As IR detectors, the spectral extension and dark current level can be controlled independently through the parameters of Δ and δE_V_, as labeled in Figure 1d. Although the dark current level can be decreased without changing the threshold wavelength by increasing Δ while maintaining the same δE_V_ (and thus, Δ′), the attainable Δ in the original Al_x_Ga_1-x_As is limited to 0.4 eV, beyond which a direct-to-indirect band transition occurs. Consequently, the AlGaAs sensors showed a detectivity of only ~10^5^ Jones in the 3–5 µm range at room temperature [44].
2. The extension of spectral response requires the hot-hole mean-free path to be longer than their traveling length in the absorber layer (x_2_–x_3_ region in Figure 1c,d) to prevent relaxation and effectively create the quasi-Fermi level. A narrower AlGaAs absorber design can easily fulfill this request, but the scattering on the heterojunction interfaces will introduce another bottleneck. The island growth during the GaAs/AlGaAs MBE process could also create interfacial roughness that acts as a defect scattering the hot holes [45,46], either dissipating their energy or elongating their trajectory inside the absorber, hindering the device performance.
3. The IR detectors with all the above-mentioned advantages fundamentally rely on the intra-band transition, which is enabled by the spin–orbit interaction-induced split-off band. All theoretical analysis about the quasi-Fermi level formation and dark current benefits is also based on this assumption. On the other hand, the system still lacks sufficient tunability on the split-off band in AlGaAs materials. Consequently, uncertainties about feasibility and versatility still exist in the above-introduced theoretical model.

### 4.1. The Emergence of van der Waals Quantum Materials (vdW-QMs)

**Figure 7:**
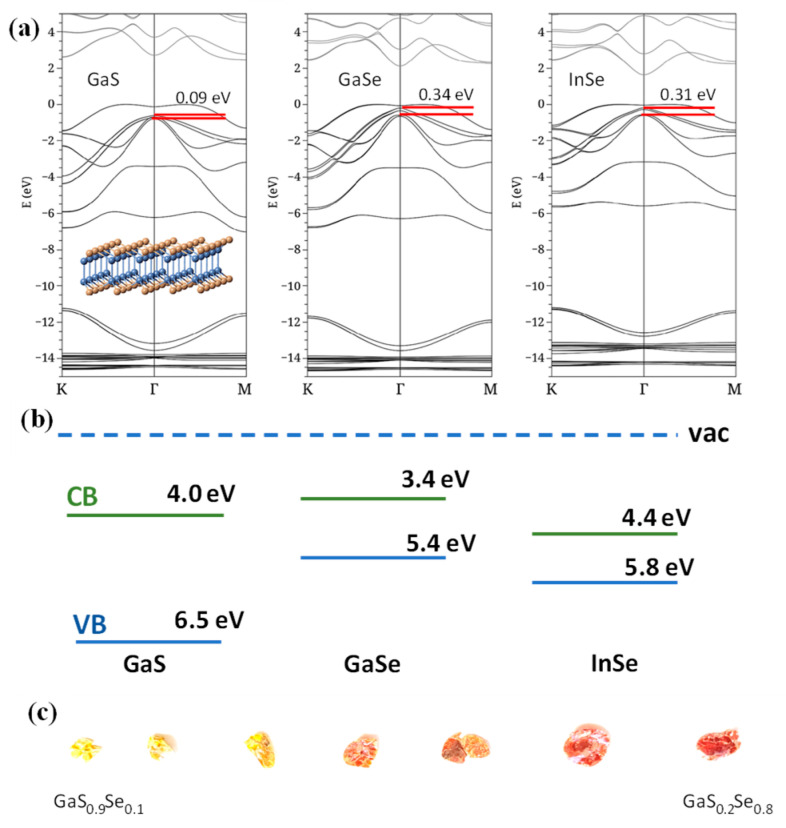
(**a**) Energy band structures (from left to right) of GaS, GaSe, and InSe: Refs. [41,42] (inset, lattice structure of these materials). (**b**) GaSexS1-x series, showing continuously varying energy band gap. (**c**) GaSxSe1-x alloy series synthesized by our team.

The above limitations in terms of constrained aluminum concentration, heterostructure interface quality, and so forth in AlGaAs-based detectors could be potentially addressed with the application of new semiconductor materials, especially with the emergence of vdW-QMs. For example, III–VI group QMs, such as GaS, GaSe, and InSe have manifold advantages; as such, we can reassemble the AlGaAs detector architecture with the above challenges addressed. (1) The QMs provide a widely tunable spin–orbit coupling strength and band split gap (Δ_SO_). As shown in Figure 7, all the listed materials have a spin–orbit interaction in their valence band and split their p_x,y_-orbitals into sub-bands. Specifically, GaSe has a Δ_SO_ of 0.34 eV [47,48], corresponding to a 3.5 µm IR response. The value can be reduced by alloying it with GaS, which has a much smaller Δ_SO_ of 0.09 eV. On the other hand, the doping of heavier elements, e.g., tellurium [49,50,51], can possibly enlarge the splitting. (2) Continuous alloying with no lattice or band structure change can be carried out. Many QMs share similar lattice structures. Furthermore, the above-listed materials also have similar energy band structures. Therefore, people can synthesize alloys among them with arbitrary ratios without worrying about the structural change encountered in the AlGaAs system. Figure 7c illustrates the GaSe-GaS alloys with continuously varying band gaps. Therefore, it is reasonable to expect that we will be able to control Δ_SO_, the band offset (Δ), and δE_V_ in a broader range than in AlGaAs. By doing so, we can better block the dark current without changing the lattice or electronic structures, and thus, improve the detectivity of the sensor in the 3~5 µm window. (3) The self-passivated atomically flat vdW-QMs surfaces can render smoother heterojunction interfaces. All vdW-QMs have layered crystal structures and self-passivated surfaces. This feature drastically simplifies the heterostructure fabrication with no concerns about matching lattice constants or thermal expansion rates. Their ultra-thinness and dangling-bond-free interfaces further allow us to optimize the absorber thickness, minimizing the interfacial scattering and hot-hole thermalization in the absorber, and thus increasing the operating temperature. With all of these benefits, vdW-QMs and their heterostructures could serve as a building block of the next-generation IR sensing materials. To date, several attempts have been made in this direction.

### 4.2. The Design of vdW-QMs Based Heterostructures

In order to implement vdW-QM-based IR detectors, it is necessary for us to first investigate the feasibility to reassemble the AlGaAs architecture. Similar to the split-off band in GaAs, theoretical calculations [47,48] indicate that the III–VI vdW-QMs (including GaS, GaSe, InSe, and their alloys) have spin–orbit splitting (Δ_SO_) ranging from 0.09 to 0.34 eV, as illustrated in Figure 7a,b. Their valence band maxima are located at 6.5 eV, 5.4 eV, and 5.8 eV, respectively [52,53,54], meeting the requirements of the IR sensing heterostructures. More importantly, these materials share the same lattice structure and similar electronic structures, as illustrated in Figure 7a inset, allowing us to tune Δ_SO_, Δ, and δEv in a broader range than that in AlGaAs without encountering direct-to-indirect band transition. The new heterostructure is expected to better suppress the dark current and increase the working temperature. For instance, the InGaSe system can provide a Δ of 0.7 eV, while the GaSeS system can yield a Δ up to 1.1 eV. Therefore, both systems can provide a much higher Δ value than AlGaAs, and better suppress the dark current as indicated by Equation (1). In the meantime, we can tune δEv independently to form a quasi-Fermi level (Δ′) around 0.25 eV and create an IR extension to 5 µm.

**Figure 8:**
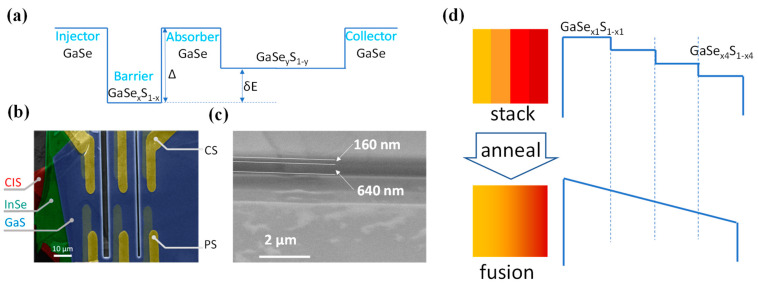
(**a**) Valence band alignment of a proposed GaSe-based asymmetric IR detector structure. (**b**) False-color SEM image of a vdW-QM heterostructure. (**c**) Its cross-section: Ref. [50]. (**d**) Procedure of graded injection barrier construction.

Other than the splitting, it is also important to introduce p-type doping in order to enable the intra-band excitation in the valence band. Early studies have already demonstrated that arsenic or phosphorus [55] doping during single crystal growth can implement this purpose. By tuning the GaS to GaSe ratio, it is possible to continuously tune parameters of Δ and δE, and fabricate the structure shown in Figure 8a. Since GaS and GaSe have an ionization energy (valence band maximum to vacuum) of 6.5 eV and 5.4 eV, respectively; the largest achievable Δ is 1.1 eV [52]. Assuming a linear variation as a function of the S-to-Se ratio, which is based on experimental confirmation, a junction in a configuration of GaSe-GaSe_0.6_ S_0.4_-GaSe-GaSe_0.5_S_0.5_ renders a Δ of 0.4 eV, and a δE of 0.1 eV, comparable with our SP1001 device. Devices with other Δ and δE values will also be fabricated in our study to find the proper combinations for dark current control and spectral extension at room temperature. Because a much higher Δ can be achieved in the GaSeS junction than its AlGaAs counterpart, a much lower dark current level according to Equation (1) is expected, and thus a higher operating temperature.

With respect to the fabrication process, the well-developed dry-transfer technique provides a proven procedure for heterostructure structure construction with ultra-high interface quality, as verified by many pioneering studies, particularly on high-performance microelectronics device fabrication. Recently, Lei et al. employed this transfer technique to fabricate a vertical color sensor architecture that exhibits multiple-band photoresponse and excellent heterojunction interfaces, as demonstrated in Figure 8b,c [56].

Another interesting device configuration replaced the flat injector barrier with a graded barrier to boost the photoresponsivity, as illustrated in Figure 6a [36]. Obviously, such a structure cannot be obtained with only the dry-transfer and stacking procedure. Instead, it is possible to build it in the manner shown in Figure 8d. Several GaSe_x_S_1-x_ pieces with different Se-to-S ratios are stacked and annealed below the melting temperature (<960 °C) to induce infusion and deliver a graded structure.

### 4.3. Dark Current Suppression

**Figure 9:**
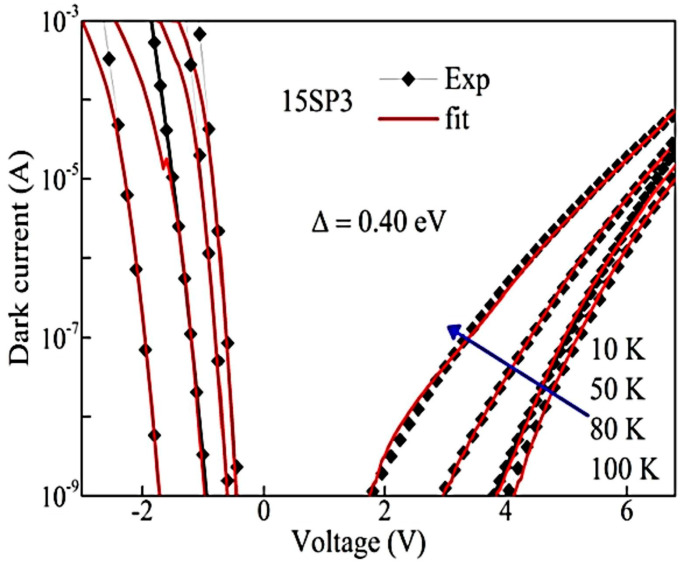
Temperature-dependent dark currents of GaAs-based device 15SP3. The theoretical fittings indicate the device always show a potential barrier of 0.40 eV, independent of temperature or formation of a quasi-Fermi level.

Compared with conventional IR detectors, the unique advantage of asymmetric heterostructure design is the separation of photoexcitation and dark current. The photoexcitation, particularly the extended IR response, explicitly depends on the formation of a quasi-Fermi level Δ′ that is determined by δE_V_. The dark current level, on the other hand, is decided by Δ. Indeed, recent temperature-dependent dark current measurements on the sample 15SP3 and theoretical fittings (Figure 9) agree well with the designed value of Δ = 0.40 eV, instead of the effective threshold Δ′ which varies from 0.027 eV to 0.40 eV. This observation, again, confirms that we will be better able to suppress the dark current by increasing Δ while sustaining the IR extension. However, as mentioned earlier, the attainable Δ in the original Al_x_Ga_1-x_As is limited to 0.4 eV, beyond which a direct-to-indirect band transition occurs. This bottleneck is readily overcome by the III–VI layered semiconductors, due to their similar band structures. GaSe/GaS_x_Se_1-x_ junctions can potentially deliver a wider Δ range, while maintaining a constant δE_V_. Considering that $I_{dark}\propto exp(-\Delta /k_{B}T)$, we estimate that the GaSeS-based sensor reduces 96% of the dark current generated by the AlGaAs-sensor. In other words, the room-temperature dark current level of the GaSeS sensor will be equal to that of an AlGaAs sensor around 130 K.

### 4.4. Formation of Quasi-Fermi Level and IR Extension at Room Temperature via Structural Optimization

The implementation of the above GaSSe structures can potentially reassemble the AlGaAs heterostructure but with a broader tuning range of Δ and δE. As such, this system is expected to better suppress the dark current and increase the operating temperature for IR sensing. On the other hand, δE_V_ is not the only factor determining the formation of a quasi-Fermi level and IR response extension. In competition with the hot-hole injection and quasi-Fermi level formation is hot-hole relaxation, which, in turn, hinders the stable existence of such an energy level. This detrimental thermal effect has been observed in our GaAs-based devices, as illustrated in Figure 4, which indicates the disappearance of IR extension as temperature increases.

One primary reason for the hot-hole relaxation is hole–photon scattering. As a rough theoretical analysis, we consider hole–phonon scattering as the primary relaxation mechanism and accordingly speculate that the mean-free-path is inversely propositional to temperature in a wide range. In other words, the device’s working temperature should increase linearly as the barrier thickness decreases at both high-temperature and low-temperature extremes. At high temperatures, the scattering rate is simply proportional to the phonon number in the term of $n\propto \exp\left(-\frac{E_{ph}}{kT}\right)\approx kT/E_{ph}$. Thus, the lifetime is τ∝1/T, and the mean-free-path is proportional to 1/T. In the case of low temperature, hot holes are distributed significantly further away from the equilibrium and occupy highly excited states far above the (quasi-) Fermi level, and the hot-hole relaxation is dominated by the phonon-emission process, in which the electron (hole)–phonon coupling coefficient is proportional to temperature T, so that the mean-free-path is also proportional to 1/T. More sophisticated first-principles models also confirm our estimation of this linear temperature dependence [57]. Thus, reducing the absorber thickness turns out to be an effective way to minimize the hot-hole relaxation. In other words, it is expected that the IR detector operating temperature increases linearly with a thinner absorber thickness. The ultra-flatness of the vdW-QM cleavage allows people to freely adjust the absorber thickness and justify this picture. The relationship between the working temperature and absorber thickness will be built to better explain the origin of the quasi-Fermi level.

It is worth mentioning that reducing the absorber thickness by half can always double the operating temperature, whereas it will not necessarily decrease the IR absorption drastically—considering the exponent in the Lambert–Beer law. For example, if the absorber thickness is reduced from twice to one times the absorption length, the absorption decreases by 27%. This indicates that it is possible to balance operating temperature and IR detection by optimizing the absorber thickness.

## 5. Conclusions

Heterojunction structures have been experimentally verified for IR sensing with an extended threshold wavelength beyond the limit defined by the standard energy gap [15], whereas their dark current is limited by a much shorter wavelength. However, the observed operating temperature was still not limited by the larger energy gap, indicating a dependence on the carrier lifetime. The new QM-based IR sensor could have a room-temperature detectivity (D*) comparable with or even exceeding the value previously achieved around 130 K by our AsGaAs-based counterpart, i.e., ~10^8^ Jones [17]. The success of this project will show a tunable Δ_SO_ in GaS_x_Se_1−x_ and In_x_Ga_1−x_Se as a function of x, establishing their quantitative relationship. This will allow one to widely tune δE and reassemble the asymmetric IR sensing structure with the newly proposed vdW-QMs, obtaining an IR response tunable to match the peak in the atmospheric window. The alloying of these material candidates should also exhibit a continuously varying valence band maxima offset (Δ) up to 1.1 eV (on GaSe/GaS heterojunction interface), without encountering lattice structure change or direct-to-indirect energy band change. This will allow us to better suppress the dark current at room temperature [39]. Compared with conventional heterostructure fabrication techniques, such as MBE, the transfer method facilitates high-quality vdW-QM structure fabrication with atomically flat interfaces and atom-level junction thickness, eliminating the concern of lattice constant matching. The success of the project will render high-quality absorption layers with a thickness less than the hot-hole mean-free-path at room temperature. Along with that, the ultra-flat interface will also minimize defect scattering encountered in AlGaAs interfaces due to island growth during MBE growth. Both benefits will render a more robust quasi-Fermi level, and thus, stabilize the IR response extension. For example, by matching the absorber width with the hot-hole mean-free-path, we expect that at room temperature the GaSeS sensor will yield a similar responsivity level to that of AlGaAs around 80 K (See Figure 4). The newly observed graphene-induced interfacial states and resonant tunneling can narrow down the thermal distribution of injected charge carriers, and thus cool down their effective temperature, even if the lattice is at room temperature. The successful introduction of this graphene tunneling junction should increase the signal-to-noise ratio and detectivity of the detector, compared to the heterostructure design. The accomplishment of these aspects will deliver a novel IR sensing mechanism, expand the material selection scope, and boost the IR sensing capability at room temperature. Compared with other IR sensing architectures working at a 3~5 µm atmospheric window, the new configuration will have much higher operating temperatures, and thus fewer auxiliary setups and a more compact volume particularly suitable for a breadth of applications including but not limited to defense, telecommunication, environmental surveillance, and security.

## Figures and Tables

**Table 1:** Device parameters for several symmetric split-off detector samples. p-doping, the Al mole fraction, the standard activation energy Δ associated with the dark current (Equation (1)), and the corresponding threshold wavelength λ_t_ and effective activation energy Δ′ from the experimental spectral response λ_eff_ are also listed. For conventional devices, λ_t_ ~ λ_eff_ (within the experimental uncertainties)—indicating no hot-carrier energy transfer. The thickness of the absorber in HE0204 is 120 nm, whereas for SP1, SP2, and LH1002 it is 18.8 nm. The reported spectral response measured at 50 K and the threshold wavelength (λ_t_) of response for all samples were determined by temperature-dependent internal photoemission spectroscopy (TDIPS) fitting [35,36].

No.	Sample	p-Doping (cm^−3^)	Al Mole Fraction x_1_ = x_2_ = x_3_ = x_4_				Δ (eV)	λ_t_ (µm)	Δ′ (eV)	λ_eff_ (µm)
A	HE0204	1 × 10^18^	0.12	0.12	0.12	0.12	0.077	16.1	0.077	16.1
M	Modeled	1 × 10^19^	0.22	0.22	0.22	0.22	0.091	13.7	0.091	13.7
B	SP1	3 × 10^18^	0.28	0.28	0.28	0.28	0.157	7.8	0.144	8.2
C	SP2	3 × 10^18^	0.37	0.37	0.37	0.37	0.211	5.9	0.190	6.0
D	LH1002	1 × 10^19^	0.57	0.57	0.57	0.57	0.30	4.13	0.295	4.2

**Table 2:** Sample details used to study the effect of the gradient and barrier energy offsets. Device parameters and the corresponding conventional activation energy (Δ), threshold wavelength (λ), effective activation energy (Δ′), and effective threshold wavelength (λeff) at 50 K are listed. The thickness of the absorber is 80 nm for all three samples. For SP1001, the spectral photoresponse at 5.3 K is also shown, since at 50 K, λeff is close to λt.

Sample	p-Doping (cm^−3^)	Al Mole Fraction				δE_v_ (eV)	Δ (eV)	λ_t_ (μm)	Δ′ (eV)	λ_eff_ (μm)
		x_1_	x_2_	x_3_	x_4_					
SP1001	1 × 10^19^	0.75	0.75	0.57	0.57	0.10	0.40 0.40	3.1 3.1	0.302 0.034	4.1 at 50 K ~36 at 5.3 K
SP1007	1 × 10^19^	0.45	0.75	0.57	0.57	0.10	0.40	3.1	0.139	8.9
15SP3	1 × 10^19^	0.45	0.75	0.39	0.39	0.19	0.40	3.1	0.090	13.7
